# Effects of fractional laser combined with amorolfine hydrochloride cream on fungal clearance rate and nursing outcomes in patients with onychomycosis: a retrospective clinical study

**DOI:** 10.3389/fmed.2026.1869163

**Published:** 2026-08-14

**Authors:** Dan Wang, ZhuoYan Wu, Fei Jia

**Affiliations:** Department of Dermatology, Xijing Hospital of The Fourth Military Medical University, Xi'an, China

**Keywords:** amorolfine hydrochloride cream, effect, fractional laser, fungal clearance rate, nursing care, onychomycosis

## Abstract

**Background/objectives:**

To investigate the clinical efficacy, fungal clearance rate, and impact on quality of life of fractional laser combined with amorolfine hydrochloride cream in the treatment of onychomycosis.

**Methods:**

This study retrospectively analyzed 90 patients diagnosed with onychomycosis who were admitted to our hospital between November 2023 and November 2024. According to the treatment strategies received during routine clinical practice, patients were assigned to either the conventional group or the study group, with 45 cases in each group. The conventional group received amorolfine hydrochloride cream alone combined with routine nursing care, whereas the study group additionally received fractional laser therapy and targeted comprehensive nursing intervention on this basis. Comparative analyses were conducted between the two groups with respect to the fungal clearance rate, overall clinical intervention efficacy, quality-of-life scores before and after the intervention, incidence of adverse events, and nursing satisfaction.

**Results:**

After the intervention, the overall clinical effective rate and fungal clearance rate in the study group (95.56 ± 3.5, 95.56 ± 3.2%) were both significantly higher than those in the conventional group (82.22 ± 4.2, 77.78 ± 4.9%) (*p* < 0.05). The degree of improvement in quality of life among patients in the study group was significantly better than that in the conventional group (*p* < 0.05). In addition, the incidence of adverse events in the study group was significantly lower than that in the conventional group (*p* < 0.05), and the overall patient satisfaction with nursing care was significantly higher than that in the conventional group (*p* < 0.05).

**Conclusion:**

Compared with amorolfine hydrochloride cream alone, fractional laser combined with amorolfine hydrochloride cream is safer and more effective in the treatment of onychomycosis, improves nursing outcomes, increases the fungal clearance rate, enhances quality of life, and reduces adverse events.

## 1 Introduction

Onychomycosis, also known as tinea unguium, is a chronic fungal infection of the nail apparatus caused mainly by dermatophytes, yeasts, and non-dermatophyte molds (1, 2). It represents the most common nail infection worldwide and accounts for a substantial proportion of superficial fungal infections, with an estimated global prevalence of approximately 4% in the general population (3). The incidence of onychomycosis has continued to increase in recent years, largely driven by population aging, the rising prevalence of diabetes mellitus and immunosuppressive conditions, and lifestyle-related risk factors (4, 5). Clinically, onychomycosis is characterized by nail thickening, discoloration, brittleness, deformity, and subungual hyperkeratosis, which can impair nail appearance, physical comfort, and overall quality of life. Although not life-threatening, its chronic course and high recurrence rate result in a substantial clinical and socioeconomic burden (6). Therefore, effective treatment strategies that improve fungal clearance and reduce recurrence are urgently needed.

In clinical practice, care is often administered using amorolfine hydrochloride cream therapy. Amorolfine hydrochloride cream is a broad-spectrum antifungal agent that exerts a potent inhibitory effect on fungi in the nail bed (7). However, because the nail plate is a dense keratinized structure with limited drug permeability, topical amorolfine monotherapy often requires long treatment periods and may achieve suboptimal fungal clearance rates (8). Therefore, approaches that enhance drug penetration and improve therapeutic efficacy are urgently needed.

Fractional CO₂ laser therapy has recently emerged as an adjunctive treatment option for onychomycosis. The laser creates controlled microthermal channels within the nail plate, which improves nail permeability and facilitates the delivery of topical antifungal agents to deeper infected sites. Meanwhile, its photothermal effect contributes to fungal reduction and modulation of local inflammatory responses, promoting nail plate repair and regeneration (9, 10). This method not only demonstrates a certain therapeutic effect in improving nail appearance and alleviating pain and pruritus, but also shortens the care cycle and reduces the fungal load, yielding excellent care outcomes (11, 12). Therefore, combining fractional CO₂ laser therapy with amorolfine hydrochloride cream may provide complementary advantages, with laser treatment improving drug delivery and fungal elimination, while amorolfine maintaining sustained antifungal activity against residual pathogens.

However, clinical evidence regarding the effectiveness and comprehensive nursing outcomes of fractional CO₂ laser combined with amorolfine hydrochloride cream remains limited. Most previous studies have focused primarily on mycological cure rates, whereas patient-centered outcomes, including quality of life improvement, treatment satisfaction, and adverse reactions, have received insufficient attention. Therefore, we hypothesized that fractional CO₂ laser combined with amorolfine hydrochloride cream would provide superior clinical benefits compared with amorolfine hydrochloride cream alone in patients with onychomycosis. Therefore, this retrospective comparative study was conducted to evaluate whether fractional CO₂ laser combined with amorolfine hydrochloride cream was associated with improved clinical outcomes compared with amorolfine hydrochloride cream alone. We hypothesized that combination therapy may enhance fungal clearance, improve quality of life, and maintain an acceptable safety profile.

## 2 Materials and methods

### 2.1 Study object

**Figure 1:**
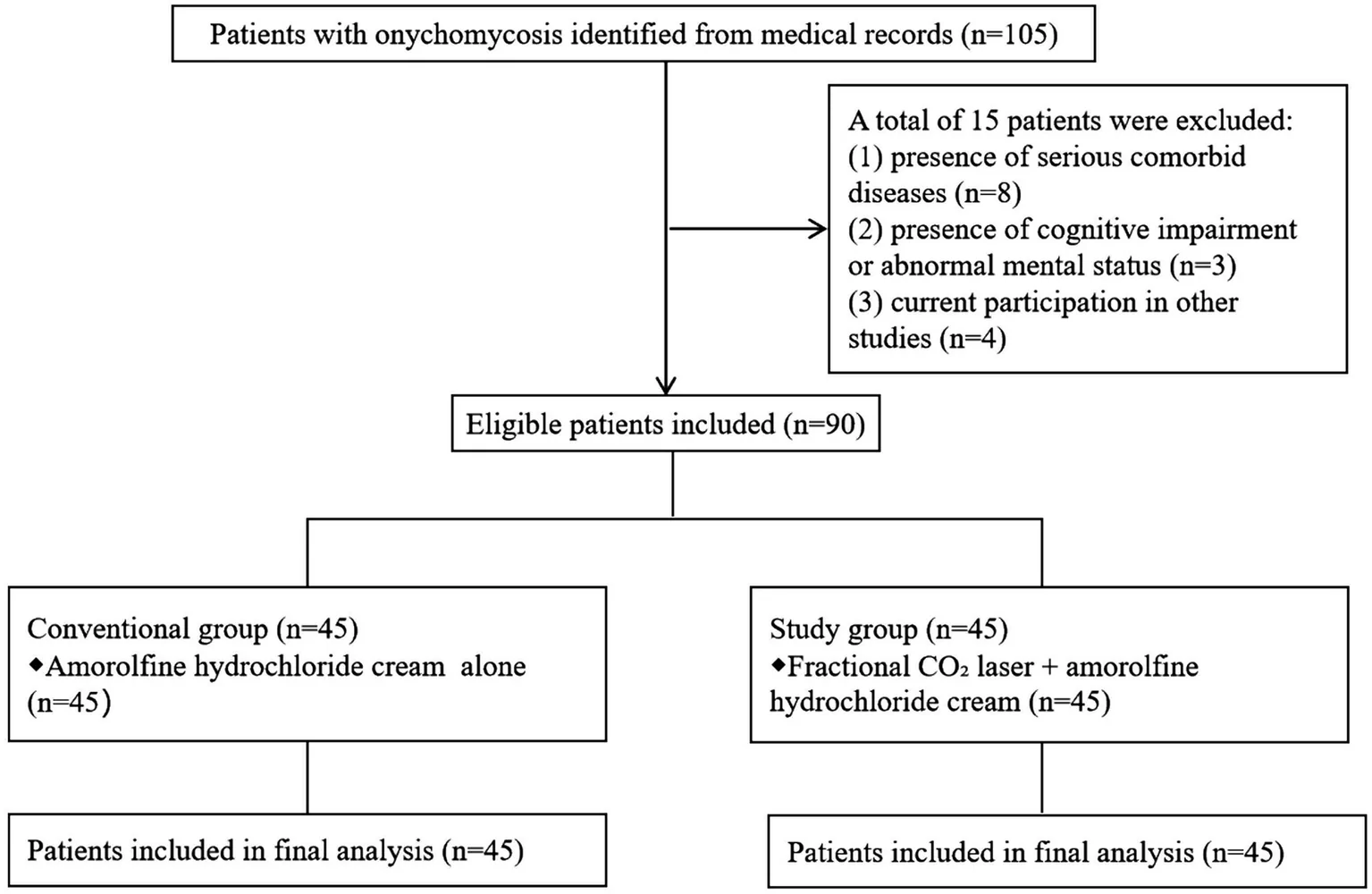
Flow chart of patient selection.

A total of 90 patients with onychomycosis, admitted to our hospital from November, 2023 to November 2024, were enrolled and randomly assigned to two groups (*n* = 45). Conventional group: treated with amorolfine hydrochloride cream alone; study group: fractional laser + amorolfine hydrochloride cream. Inclusion criteria: (1) patients who met the diagnostic criteria for onychomycosis (13); (2) patients with complete clinical data; and (3) patients who were informed of the study content and agreed to participate. Exclusion criteria: (1) presence of serious comorbid diseases; (2) presence of cognitive impairment or abnormal mental status; (3) current participation in other clinical studies (Figure 1).

### 2.2 Methods

Patients were categorized according to the treatment strategies received during routine clinical practice. The study group received the same treatment and routine nursing care as the conventional group, with the addition of fractional CO₂ laser therapy. Before each laser session, the affected nails were soaked in warm water for approximately 30 min to soften the nail plate, followed by trimming of detached or thickened nails and gentle filing to reduce nail thickness. Nail debris was then removed with warm water or sterile saline before laser treatment.

Fractional CO₂ laser treatment was performed using a pulse energy of 100–150 mJ and a spot spacing of 0.3–0.6 mm. The scanning area was adjusted according to the size of the affected nail and extended approximately 2–3 mm beyond the visible lesion margin. Each lesion received 2–6 scanning passes, and treatment was continued until a mild tolerable burning sensation was achieved. Laser parameters could be adjusted slightly according to nail thickness, lesion size, and individual patient tolerance while remaining within the recommended treatment range. Laser treatment was administered once every 2 weeks for a total of 3–6 sessions, depending on the severity and extent of the nail lesions. During each treatment session, both patients and medical staff wore appropriate laser protective eyewear.

Immediately after laser treatment, the nail bed was disinfected and 5% amorolfine hydrochloride cream was applied topically, followed by a simple sterile dressing when necessary. Patients were instructed to continue home-based nail care, including warm-water soaking, trimming detached nails, thinning the nail plate with a nail file, removal of nail debris, topical application of amorolfine hydrochloride cream, and occlusive wrapping of the treated nail with plastic film for approximately 2 h after medication application to enhance drug penetration.

### 2.3 Observational index

All outcome measures were evaluated at the completion of the treatment course.

#### 2.3.1 Nursing effects

The nursing effects were primarily classified as markedly effective (the affected nail completely returned to a healthy condition), effective (more than 60% of the affected nail area returned to a healthy condition), and ineffective (the above criteria were not met). Total effective rate = (markedly effective + effective)/45 × 100.00%.

#### 2.3.2 Fungal clearance rates

Comparison of fungal clearance rates between the two patient groups.

#### 2.3.3 Quality of life

According to the GQOLI-74 scoring scale, a comparative analysis was conducted of patients’ quality of life before and after nursing care. The scale comprises psychological function (20–100 points), physical function (20–100 points), material life (16–80 points), and social function (20–100 points). Higher scores indicate better quality of life.

#### 2.3.4 Adverse event occurrence

Treatment-related adverse events were retrospectively reviewed based on clinical records during the treatment and observation period. The evaluated adverse events included periungual erythema, periungual dryness, and periungual burning sensation. In addition, other clinically significant adverse events associated with fractional CO₂ laser therapy, including infection, scarring, and persistent nail plate damage, were reviewed. The incidence of adverse events was compared between the two groups.

#### 2.3.5 Satisfaction

Patient satisfaction in the two groups was analyzed in detail using a satisfaction scale, which included three categories: very satisfied, satisfied, and dissatisfied. The overall satisfaction rate was calculated as follows: overall satisfaction = (very satisfied + satisfied) / n × 100.00%. ① Very satisfied (80–100 points). ② Satisfied (60–79 points). ③ Dissatisfied (below 60 points).

### 2.4 Statistical analysis

SPSS 23.0 software was used for data collection, organization, and analysis.% were used to express count data, and the χ^2^ test was employed to assess differences between the two groups. Measurement data were expressed as ( $\bar{x}$ ± s), and the t-test was used to evaluate differences between the two groups. If a difference was observed between the two groups, this indicated *p* < 0.05; if no difference was observed, then *p* > 0.05.

## 3 Results

### 3.1 Baseline characteristics between the two groups of patients

A total of 90 patients with onychomycosis who met the inclusion criteria were included in this retrospective study. According to the treatment received in routine clinical practice, patients were categorized into the Study group (*n* = 45) and the conventional group (*n* = 45). As shown in Table 1, there were no statistically significant differences in age or sex between the two groups (*p* > 0.05). Onychomycosis commonly occurred in the fingernails and toenails, with no significant difference between the two groups (*p* > 0.05). There were also no statistically significant differences between the two groups in the lesion subtypes SWO, DLSO, and PSO (*p* > 0.05).

**Table 1:** Baseline characteristics.

Group	Study Group (*n* = 45)	Conventional group (*n* = 45)	χ^2^/t	p
Age (years)	34.34 ± 1.36	34.77 ± 1.65	1.349	0.181
Sex			0.178	0.673
Male	22	24		
Female	23	21		
Type of diseased nails			0.194	0.660
Nail	15	17		
Toenail	30	28		
Disease course (months)	7.54 ± 2.41	7.92 ± 2.73	0.700	0.486
Lesion classification			0.306	0.909
SWO	10	8		
DLSO	28	29		
PSO	7	8		

SWO, superficial white onychomycosis; DLSO, distal and lateral subungual onychomycosis; PSO, proximal subungual onychomycosis.

### 3.2 Nursing efficacy

The overall nursing effectiveness rate in the study group was higher than that in the conventional group, with a statistically significant difference between the groups (*p* < 0.05) (Table 2).

**Table 2:** Nursing efficacy (*n*, %).

Group	Markedly effective	Effective	Ineffective	Total effective rate (%)
Study group (*n* = 45)	35 (77.78)	8 (17.78)	2 (4.44)	43 (95.56)
Conventional group (*n* = 45)	25 (55.56)	12 (26.67)	8 (17.78)	37 (82.22)
χ^2^	-	-	-	4.050
P	-	-	-	0.044

### 3.3 Comparison of fungal clearance rates

The fungal clearance rate in the study group was higher than that in the conventional group, with a statistically significant difference between the groups (*p* < 0.05) (Table 3).

**Table 3:** Comparison of fungal clearance rates between the two patient groups (%).

Group	Complete clearance	Not cleared	Overall clearance rate (%)
Study group (*n* = 45)	43(95.56)	2(4.44)	43(95.56)
Conventional group (*n* = 45)	35(77.78)	10(22.22)	35(77.78)
χ^2^	-	-	6.153
P	-	-	0.013

### 3.4 Quality of life assessment

The scores for all quality-of-life dimensions in the study group were higher than those in the conventional group, with a statistically significant difference between the groups (*p* < 0.05) (Table 4).

**Table 4:** Assessment of quality of life between groups ( $\bar{x}$ ±s).

Group	Psychological function		Physical function		Material life		Social function	
	Before nursing	After nursing	Before nursing	After nursing	Before nursing	After nursing	Before nursing	After nursing
Study group (*n* = 45)	45.32 ± 1.24	66.56 ± 1.22	46.22 ± 1.32	67.34 ± 4.22	45.12 ± 1.23	63.32 ± 4.25	47.35 ± 1.23	67.64 ± 7.25
Conventional group (*n* = 45)	45.24 ± 1.25	59.76 ± 1.24	46.13 ± 1.24	58.25 ± 7.23	45.13 ± 1.22	57.23 ± 7.21	47.43 ± 1.22	60.31 ± 7.24
t	0.304	26.222	0.333	8.316	0.038	4.881	0.309	4.799
P	0.761	0.000	0.739	0.000	0.969	0.000	0.757	0.000

### 3.5 Incidence of adverse events

The overall incidence of adverse events in the study group was lower than that in the conventional group, and the difference between the groups was statistically significant (*p* < 0.05) (Table 5). No severe or unexpected treatment-related adverse events, including infection, scarring, or persistent nail plate damage, were identified in the medical records during the observation period.

**Table 5:** Incidence of adverse events (*n*, %).

Group	Periungual erythema	Periungual dryness	Periungual burning sensation	Total effective rate (%)
Study group (*n* = 45)	0 (0.00)	1 (2.22)	1 (2.22)	2 (4.44)
Conventional group (*n* = 45)	3 (6.67)	3 (6.67)	3 (6.67)	9 (20.00)
χ^2^	-	-	-	5.074
P	-	-	-	0.024

### 3.6 Assessment of nursing satisfaction

The nursing satisfaction rate in the study group was higher than that in the conventional group, and the difference between the two groups was statistically significant (*p* < 0.05) (Table 6).

**Table 6:** Assessment of nursing satisfaction (*n*, %).

Group	Very satisfied	Satisfied	Dissatisfied	Overall satisfaction (%)
Study group (*n* = 45)	30 (66.67)	14 (31.11)	1 (2.22)	44 (97.78)
Conventional group (*n* = 45)	24 (53.33)	15 (33.33)	6 (13.33)	39 (86.67)
χ^2^	-	-	-	3.872
P	-	-	-	0.049

## 4 Discussion

Onychomycosis is a common chronic fungal infection of the nail that can cause nail discoloration, thickening, deformity, and impaired quality of life. Although not life-threatening, its prolonged treatment duration and high recurrence tendency remain major clinical challenges (14, 15). Therefore, improving fungal clearance while minimizing treatment-related burden is an important goal in clinical management.

The principal advantages of amorolfine hydrochloride cream therapy include broad-spectrum antifungal activity, good safety profile, convenient administration, and favorable patient compliance (16). However, because of the limited permeability of the nail plate, topical amorolfine monotherapy often requires prolonged treatment and may achieve suboptimal fungal clearance (17, 18). Therefore, therapeutic strategies that improve drug delivery and enhance antifungal efficacy are clinically valuable. Fractional CO₂ laser has emerged as a potential adjunctive therapy for onychomycosis. By generating controlled microthermal channels within the nail plate, fractional CO₂ laser can improve nail permeability and facilitate penetration of topical antifungal agents into deeper infected regions (19). In addition, its photothermal effects may contribute to fungal reduction and local tissue remodeling while minimizing damage to surrounding tissues (20, 21). Therefore, the combination of fractional CO₂ laser and amorolfine hydrochloride cream may provide complementary therapeutic effects by enhancing drug delivery and improving fungal eradication. In the present study, we demonstrated that fractional CO₂ laser combined with amorolfine hydrochloride cream significantly improved clinical efficacy, fungal clearance rate, quality of life, and nursing satisfaction compared with amorolfine hydrochloride cream alone. These findings suggest that the combined therapeutic strategy provides additional clinical benefits beyond topical antifungal therapy alone. The improved fungal clearance observed in the combined treatment group may be attributed to the ability of fractional CO₂ laser to disrupt the dense keratin structure of the nail plate, enhance drug penetration into deeper infected areas, and exert direct photothermal effects on fungal components. This complementary mechanism may overcome the limited permeability of topical amorolfine hydrochloride and contribute to more effective fungal eradication.

Previous studies have reported that fractional CO₂ laser combined with topical antifungal agents can improve clinical outcomes in patients with onychomycosis, which is consistent with our findings (22). This study found that the quality-of-life score of patients in the study group was higher than that in the conventional group (*p* < 0.05), indicating that the use of fractional laser combined with amorolfine hydrochloride cream in patients with onychomycosis improve their quality of life. Analysis of the reasons: this combination can effectively eliminate fungal infection, attenuate the inflammatory response, improve the appearance of the nails, and help patients regain confidence. The rapid onset of therapeutic effects and individualized adjustments during the nursing process can also reduce patients’ suffering and inconvenience, thereby enhancing the care experience. Furthermore,by reducing the recurrence rate of fungal infections and lowering the risk of drug resistance, it is possible to avoid the burden associated with long-term repeated interventions, thereby further improving patients’quality of life (22).

Šveikauskaitė and Shetty et al. (8, 12) demonstrated that fractional CO2 laser combined with amorolfine hydrochloride cream has significant therapeutic efficacy in the treatment of onychomycosis, which is consistent with the results of the present study. In this study, the nursing satisfaction, fungal clearance rate, and nursing efficacy in the study group were all higher than those in the conventional group (*p* < 0.05). These results indicate that, in patients with onychomycosis, treatment with fractional laser combined with amorolfine hydrochloride cream can significantly improve nursing satisfaction and the fungal clearance rate. Analysis of the reasons: this combined care regimen integrates the highly effective sterilizing effect of fractional laser therapy with the local intervention effect of amorolfine hydrochloride cream, thereby enabling more comprehensive eradication of fungal nail infection and improving the effectiveness of care. During the course of care, patients can perceive improvements in nail appearance and alleviation of symptoms, which enhances their confidence in the care and their satisfaction. Meanwhile, this combined care regimen can reduce the recurrence rate of fungal infection, lower the risk of drug resistance, improve the fungal clearance rate, and effectively prevent care failure and repeated infection. Therefore, fractional laser combined with amorolfine hydrochloride cream has a significantly positive effect on improving patient satisfaction and fungal clearance rate, thereby providing better therapeutic outcomes for patients (12, 23).

Zhou et al. (24) reported that, during the treatment of onychomycosis with fractional CO2 laser combined with 1% luliconazole cream, some patients experienced mild pain during the laser procedure, but no bleeding occurred. No adverse reactions were observed during the follow-up period. In this study provided support for the present study. In present study, the overall incidence of adverse events in the study group was lower than that in the conventional group (*p* < 0.05), indicating that nursing care for patients with onychomycosis receiving fractional laser combined with amorolfine hydrochloride cream can significantly reduce the incidence of adverse events. Analysis of the reasons: the noninvasive characteristics of fractional laser reduce damage to the periungual skin during treatment, thereby avoiding the occurrence of periungual erythema. Amorolfine hydrochloride cream, as a topical external therapy, can effectively eradicate fungi and reduce fungal irritation to the skin around the nail, thereby lowering the incidence of periungual dryness and burning sensation. Therefore, fractional laser combined with amorolfine hydrochloride cream therapy has a significantly positive effect on reducing the incidence of adverse events in patients, providing a safer and more effective care option for patients.

Although fractional CO₂ laser therapy is associated with higher initial treatment costs than topical amorolfine hydrochloride cream alone, patients receiving combination therapy in this study showed higher fungal clearance rates, improved clinical outcomes, better quality of life, fewer adverse events, and higher patient satisfaction compared with those receiving topical therapy alone. These observed clinical benefits may potentially reduce prolonged treatment duration, repeated ineffective interventions, and additional healthcare utilization, thereby partially offsetting the increased initial cost of laser therapy. Nevertheless, a formal pharmacoeconomic evaluation was not performed in this study. Future prospective studies incorporating cost-effectiveness analyses are warranted to further determine the economic value of this combined treatment strategy.

This study has several limitations. First, this was a single-center retrospective comparative study with a relatively small sample size, which may limit the generalizability of the findings. Second, because treatment allocation was based on routine clinical practice rather than prospective randomization, potential selection bias and confounding factors cannot be completely excluded. Third, the follow-up period was relatively short, and long-term recurrence rates of onychomycosis were not evaluated. Future multicenter prospective studies with larger sample sizes, longer follow-up periods, and standardized treatment protocols are needed to further validate the clinical effectiveness and long-term benefits of fractional CO₂ laser combined with amorolfine hydrochloride cream.

## 5 Conclusion

In summary, providing nursing care for patients with onychomycosis undergoing fractional laser combined with amorolfine hydrochloride cream therapy can improve nursing outcomes and yield better therapeutic efficacy for patients. However, prior to the implementation of nursing care, nursing staff should still conduct a comprehensive assessment and formulate an individualized nursing plan to ensure both the effectiveness of care and patient safety.

## Data Availability

The original contributions presented in the study are included in the article/supplementary material, further inquiries can be directed to the corresponding author/s.
